# Using paired-end sequences to optimise parameters for alignment of sequence reads against related genomes

**DOI:** 10.1186/1471-2164-11-458

**Published:** 2010-08-03

**Authors:** Abhirami Ratnakumar, Sean McWilliam, Wesley Barris, Brian P Dalrymple

**Affiliations:** 1 CSIRO Livestock Industries, 306 Carmody Road, St. Lucia, QLD 4067, Australia; 2 Department of Medical Biochemistry and Microbiology, Uppsala University, Box 582, 751 23 Uppsala, Sweden

## Abstract

**Background:**

The advent of cheap high through-put sequencing methods has facilitated low coverage skims of a large number of organisms. To maximise the utility of the sequences, assembly into contigs and then ordering of those contigs is required. Whilst sequences can be assembled into contigs *de novo*, using assembled genomes of closely related organisms as a framework can considerably aid the process. However, the preferred search programs and parameters that will optimise the sensitivity and specificity of the alignments between the sequence reads and the framework genome(s) are not necessarily obvious. Here we demonstrate a process that uses paired-end sequence reads to choose an optimal program and alignment parameters.

**Results:**

Unlike two single fragment reads, in paired-end sequence reads, such as BAC-end sequences, the two sequences in the pair have a known positional relationship in the original genome. This provides an additional level of confidence over match scores and e-values in the accuracy of the positional assignment of the reads in the comparative genome. Three commonly used sequence alignment programs: MegaBLAST, Blastz and PatternHunter were used to align a set of ovine BAC-end sequences against the equine genome assembly. A range of different search parameters, with a particular focus on contiguous and discontiguous seeds, were used for each program. The number of reads with a hit and the number of read pairs with hits for the two end sequences in the tail-to-tail paired-end configuration were plotted relative to the theoretical maximum expected curve. Of the programs tested, MegaBLAST with short contiguous seed lengths (word size 8-11) performed best in this particular task. In addition the data also provides estimates of the false positive and false negative rates, which can be used to determine the appropriate values of additional parameters, such as score cut-off, to balance sensitivity and specificity. To determine whether the approach also worked for the alignment of shorter reads, the first 240 bases of each BAC end sequence were also aligned to the equine genome. Again, contiguous MegaBLAST performed the best in optimising the sensitivity and specificity with which sheep BAC end reads map to the equine and bovine genomes.

**Conclusions:**

Paired-end reads, such as BAC-end sequences, provide an efficient mechanism to optimise sequence alignment parameters, for example for comparative genome assemblies, by providing an objective standard to evaluate performance.

## Background

With the availability of the so-called Next Generation Sequencing (NGS), relatively cheap high-throughput short molecule sequencing technologies such as Illumina GA and ABI SOLiD, and medium length sequencing technologies such as Roche 454 is giving non-specialist laboratories the ability to sequence large genomes. However, the large number of reads produced by these NGS technologies creates problems for the utilisation of the sequence data. In the last few years a number of new programs for the alignment of short reads, for example in the range 30-150 bases, have been described, these include Maq [1], SOAP [2] and Bowtie [3]. In general, these programs are designed for resequencing projects, where few nucleotide sequence differences are expected between the sequence reads and the reference genome.

However, many projects are likely to be low coverage skims of previously unsequenced genomes [4] possibly combining identification of SNPs with a survey of the genome sequence. The optimal design of SNP chips and effective utilisation of the chips in whole genome association analyses requires the relative order of and the distance between the SNPs and their association with genes to be known. Obtaining this information is likely to rely on comparative genomics by utilising the assemblies of related genomes to order and orientate sequence reads and contigs. The assembly of the cat genome based on Sanger sequencing used such a process to build an assembly from a 1.9 fold coverage of the genome [5]. For the cat, a combination of MegaBLAST and Blastz was used to generate the genome assembly, which utilised alignments to a number of other genomes such as human, chimpanzee, mouse, rat, dog, and bovine [5].

In recent years a wide range of different programs have emerged to complement BLAST, itself a compromise between specificity and sensitivity relative to the Smith-Waterman algorithm. True Smith-Waterman is too slow for large scale projects, but in an effort to approach its speed, sensitivity and specificity, MegaBLAST [6] and PatternHunter [7,8], amongst others, have been developed. A key to increasing the speed of the sequence alignment programs has been the utilisation of discontiguous seeds [7,9], allowing the matches to be spread over longer sequences with internal mismatches and therefore the utilisation of longer seeds for the same sensitivity. This approach has been implemented in MegaBLAST, Blastz [10] and PatternHunter amongst other programs. Using discontiguous seeds improves the specificity and sensitivity of the programs. Further innovations have included using multiple discontiguous seeds and refining the patterns of the seeds [11,12]. However, much of the analysis and comparisons of approaches have been carried out on mRNA/EST sequence sets [9,12] and not on genomic DNA which, in the eukaryotes, has quite different distributions of repeats. The new sequence alignment programs that have been developed for the alignment of sequence reads against reference genome sequences for resequencing projects (see above) do not appear to be suitable for comparative genomics approaches. For aligning medium length genomic sequence reads (150-500 bases) against related genomes, it is not immediately clear which program and which parameters would yield the best compromise between sensitivity and specificity.

Here we use the example of the analysis of the effectiveness of three widely used DNA sequence alignment programs to position ovine BES reads against the equine and bovine genome assemblies to demonstrate the utility of the approach. We use the information about the positional relationship of the end sequences of each BAC in the ovine genome to estimate the sensitivity and specificity of the methods of determining the positions in the related, but not identical genomes.

## Results and Discussion

### Alignment of ovine BAC-end sequences (BESs) to the equine genome assembly

**Figure 1:**
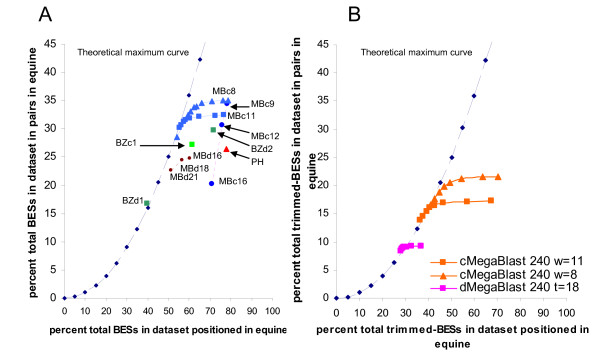
**Percentage of total ovine BESs positioned vs. percentage of total ovine BESs in tail-to-tail pairs**. A) Full length ovine BESs. B) Ovine BESs trimmed to 240 bases in length. In A) the observed results from running MegaBLAST using a contiguous word size of 8 (MBc8) are shown as blue triangles, and the observed results from running MegaBLAST with a contiguous word size of 11 (MBc11) are shown as blue squares. The transition of the megablast curves in both A) and B) from right to left show the effect of increasing the score cut-off from no score cut-off (right most symbol) to score cut-offs starting at 40 and increasing in increments of 5 up to 100 (left most symbol).

The full-length BESs from the ovine CHORI-243 BAC library, constructed from a single individual animal [13], were mapped to the draft equine genome assembly EquCab1 using MegaBLAST, Blastz and PatternHunter with a range of different parameters (Table 1). These three representative DNA sequence alignment programs were chosen to illustrate the approach for the following reasons; the BLAST suite of programs is very widely used and readily accessible to all users via the internet, Blastz underpins the multiple genome alignments calculated and displayed on the UCSC genome browser website [14], PatternHunter has been described in the published literature as approaching true Smith-Waterman performance [7,8]. The number of ovine BACs with both BESs mapped to the equine genome, in the tail-to-tail organisation and within 200 kb of each other, was calculated [13]. The BACs with their two end sequences mapped on the framework genome (equine) in the tail-to-tail organisation are the only ones with an organisation that is the same as the BAC in the original genome (ovine). The percentage of the total number of BESs positioned on the equine genome with each program and each set of parameters, and the percentage of the total number of BACs in the full dataset with BESs positioned in a tail-to-tail pair were then plotted (Figure 1A). The closer to the right of the graph the more BESs for which a position was reported (higher sensitivity), the closer to the top of the graph the greater the percentage of the total BESs in tail-to-tail pairs. The closer a point is to the theoretical curve the larger the percentage of positioned BESs in tail-to-tail pairs (higher specificity).

**Table 1:** Programs and parameters used

ID		**Word size**^**1**^	**Parameters**^**2**^
MBc16	MegaBlast	16	-r 1 -q -1 -X 40 -W 16
MBc12	MegaBlast	12	-r 1 -q -1 -X 40 -W 12
MBc11	MegaBlast	11	-r 1 -q -1 -X 40 -W 11
MBc9	MegaBlast	9	-r 1 -q -1 -X 40 -W 9
MBc8	MegaBlast	8	-r 1 -q -1 -X 40 -W 8
MBd21	MegaBlast	11/21	-t 21 -W 11 -q -3 -r 2 -G 5 -E 2 -N 2
MBd18	MegaBlast	11/18	-t 18 -W 11 -q -3 -r 2 -G 5 -E 2 -N 2
MBd16	MegaBlast	11/16	-t 16 -W 11 -q -3 -r 2 -G 5 -E 2 -N 2
BZd1	Blastz	12/19	K = 4500, L = 4500, M = 50
BZd2	Blastz	12/19	K = 2200, L = 2200, M = 50
BZc1	Blastz	8	K = 2500 L = 2500 M = 50 T = 0
PH	PatternHunter	11/18	-db 0 -mi -mj -b 2 -N 1

^1^For discontiguous searches the number of matches is listed first followed by the length of the match seed.

^2^All MegaBlast searches were run with -D 3 -U T -F 'm D'. No score filter was set to allow for subsequent filtering of matches based on scores.

PatternHunter [8] (Table 1) performed poorly, with high sensitivity, but very low specificity. This is probably due to the inability of PatternHunter to utilise "soft masking" of the repeats. That is, PatternHunter does not have the ability to initiate matches in unmasked (upper case) sequence and then extend the matches into repetitive (lower case) sequence. No further testing of PatternHunter was undertaken. As expected running MegaBLAST without soft masking also leads to significantly reduced specificity (data not shown). Thus for positioning sequences from genomes with multicopy repeats (i.e. most large eukaryote genomes) "soft masking" and associated extension options are essential for maximum specificity.

Blastz [10] has been widely used for aligning genome sequences including whole genome alignments and allows "soft masking" of the sequences. Running Blastz with a contiguous seed of length 8 bases, see BZc1 (Table 1), had a lower sensitivity than PatternHunter, with a very similar percentage of the BACs in the total dataset positioned in the tail-to-tail configuration and therefore an increased specificity, i.e. fewer BACs with positions and a higher probability that the BESs position using Blastz are correctly positioned (Figure 1A). Using Blastz with a discontiguous seed of 12 matches/19 bases, whilst keeping the remaining parameters from the previous case unchanged, returned a very similar sensitivity and specificity to search BZc1 (data not shown). Relaxing the parameters, see BZd2 (Table 1), increased the sensitivity, but with a loss of specificity (Figure 1A). Increasing the stringency of the parameters with the discontiguous seed, see BZd1 (Table 1), improved specificity to the theoretical maximum, but with a substantial penalty to the sensitivity (Figure 1A). For a detailed description of the use of seeds in sequence alignment programs see Brown 2007 [15].

MegaBLAST has been designed for very quickly searching large databases and also permits the use of contiguous and discontiguous seeds to initiate matches. The performance of discontiguous MegaBLAST at all word sizes is clearly more specific than contiguous MegaBLAST when the latter is run with no score filtering at all. With long word sizes, such as 16, discontiguous MegaBLAST is also more sensitive than contiguous MegaBLAST, but less sensitive than contiguous MegaBLAST with a word size of 12 or less. As expected, using both a seed designed for protein coding DNA and a seed designed for non-coding DNA [16] together was significantly more sensitive and slightly more specific than using a non-coding seed alone With both seeds, using 11 matches/21 base seeds and other parameters as in Table 1, 54.9% of the BESs were positioned v. 47.2% positioned with just the non-coding seed. With both the protein coding DNA and non-coding DNA seeds 21.95% of the BESs were in tail-to-tail BACS v. 18.32% positioned with just the non-coding seed. In all subsequent discontiguous MegaBLAST searches both the coding and non-coding seed were used. A comparison of alternate scoring schemes for rewards for matches and penalties for mismatches (Table 2) showed that -r, 1, -q, -1 respectively performed slightly better than -r, 2, -q, -3 for contiguous MegaBLAST, but vice versa for discontiguous MegaBLAST. All searches were run at the preferred values for the particular seed type. For discontiguous MegaBLAST a seed of 11 matches/18 bases appeared to be the best compromise between specificity and sensitivity when used with a score cut off.

**Table 2:** Effect of match reward and mismatch penalty on specificity and sensitivity of contiguous and discontiguous MegaBLAST searches

	% total BESs		% BESs positioned	Parameters	
	
	positioned	in tail-to-tail BACs	in tail-to-tail BACs	match reward, -r	mismatch penalty, -q
Contiguous^1^	76.78%	32.36%	42.15%	1	-1
	77.57%	31.91%	41.14%	2	-3
Discontiguous^2^	54.92%	21.94%	39.95%	1	-1
	51.17%	22.64%	44.24%	2	-3

^1^word size 11, with no score filtering

^2^word size of 11 with a match length of 21 using a discontiguous seed, with no score filtering

Overall, for this particular comparison, the best parameters were short contiguous word size settings for MegaBLAST. A small improvement in sensitivity over searches was observed when using a word size of 8 instead of 9 (Figure 1A). Although the increase in sensitivity versus a word size of 16 was small (from ~70% BESs positioned to ~80%) the increase in specificity was large (from ~20% in tail-to-tail pairs to ~35%).

In comparison, the virtual ovine genome approach [13], which aimed to maximise matches and by applying a filter of tail-to-tail BACs, to minimize incorrectly positioned BACs, positioned 45.5% of ovine BACs (and thus BESs) in tail-to-tail organisation on the human genome assembly. This result considerably exceeds the performance of the approaches described here, although its aim is position as many BACs as possible, not position unpaired reads, for which it is not suitable. However, this approach relied on the availability of tools for the conversion of coordinates between genome builds that may not be available for emerging genome sequences and therefore it may not be generally applicable.

### Determining the optimal score cut off

MegaBLAST search results can be filtered post completion of the searches using the alignment scores. Increasing the alignment score cut-off, substantially reduced the false positive rate with limited impact on the total number of BES for which correct positions were obtained (Table 3). Individual users will need to make the final determination of the appropriate balance between yield and specificity. This will depend on the subsequent use of the results, especially whether additional filtering steps will be used.

**Table 3:** Calculation of true and false positive and false negative rates from search results.

**Score cut-off**^**1**^	BESs with positions	BESs in tail-to-tail BACs	**BESs predicted to be in tail-to-tail BACs**^**2 **^	**fp rate**^**3**^	**fn rate**^**4**^
8	292,916	130,358	230,616	0.43	0.21
40	283,315	130,236	215,746	0.40	0.24
45	263,060	129,806	186,000	0.30	0.30
50	245,983	128,826	162,636	0.21	0.34
55	236,647	126,698	150,524	0.16	0.36
60	231,047	125,670	143,484	0.12	0.38
65	225,201	123,140	136,314	0.10	0.40
70	221,398	121,134	131,750	0.08	0.40
100	200,409	106,368	107,954	0.01	0.46

^1^results from the MegaBLAST search on full length BESs using a contiguous word size of 8 the default score cut-off is 8, the results in this row correspond to the case where no score cut-off is specified.

^2^for each score cut-off the number of BESs predicted to be in tail-to-tail BACs assuming all BESs were correctly positioned.

^3^false positive rate

^4^false negative rate

### Using medium length sequence reads

To test the applicability of the approach described here with sequences of shorter length the ovine BESs were trimmed to the first 240 bases, whilst retaining the pairing information. A smaller number of searches of program and parameter values were undertaken for the alignment of trimmed ovine BESs against the equine genome assembly (Figure 1B). As with the full length sequences, contiguous MegaBLAST performed substantially better than discontiguous MegaBLAST. In contrast to the full length BESs, where a word size of 8 was slightly better than 11, the improvement in using a word size of 8 over a word size of 11 was far greater for medium length sequences, especially once the score cut off was applied.

### Mapping medium length sequence reads to bovine genome assemblies

**Figure 2:**
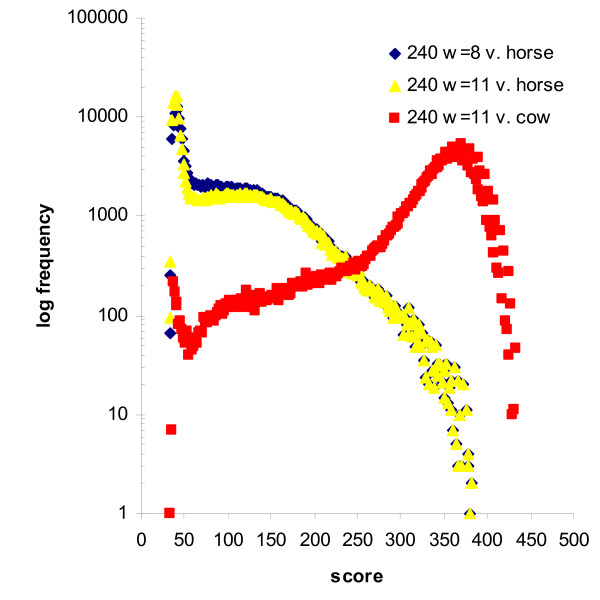
**Distribution of MegaBLAST scores for trimmed ovine BESs vs. the equine and bovine genome assemblies**.

The ovine and bovine genome sequences are more closely related to each other than either is to the equine genome. We explored the choice of parameters for aligning the trimmed ovine BESs sequence reads to the bovine genome assembly. Although contiguous MegaBLAST was also better than discontiguous MegaBLAST, the difference was not as large as that observed for the searches against the equine genome (data not shown). Thus the relative performance of the different sets of parameters is also dependent on the characteristics of the sequence datasets being used for a particular comparison. This reinforces the need for the empirical comparison of programs and parameters with the datasets to which they will be applied. The frequency distribution of the alignment scores for the 240 base trimmed BESs against the equine and bovine genomes show marked differences (Figure 2). There were large numbers of high scoring matches against the bovine genome. But as expected, the number of matches against the equine genome decreased as the score increased. However, for both comparisons a clear inflexion in the curve around a score of 55 is observed due to an increase in sequences with positions. As a result, the parameters that are optimal for the alignment of the ovine reads against the equine genome assembly appear to be similar to, if not the same as, the optimal parameters for the alignment of the ovine sequences against the bovine genome assembly. However, the additional sensitivity benefits of the short contiguous seed probably do not outweigh the increased speed of the longer discontiguous seed for alignment against the bovine genome sequence. Especially since the similarities in the repeat sequences between bovine and ovine generates extremely large numbers of hits at short word lengths (for example w = 8) even with sequences masked at the most sensitive settings of repeat masker (data not shown) rendering the use of w = 8 impractical.

Using an earlier version of the bovine genome assembly (Btau3.0) reduced the sensitivity and specificity scores for all search programs and parameter sets tested relative to Btau4.0, but did not alter the relative ranking of the programs and parameters (data not shown). Thus where the objective is to optimise search parameters, as long as the order of contigs and scaffolds in the comparison genome is approximately correct, this approach will be effective.

## Conclusions

Paired-end sequences provide a very useful dataset for optimising program and parameters for positioning sequence reads (or contigs) with a range of different lengths from one genome against another genome. Parameter estimation can be undertaken with genomes in various stages of assembly, although a substantial number of scaffolds much longer than the average length of the inserts in the paired-end reads are required. Surprisingly, MegaBLAST with contiguous seeds performs better than discontiguous seed MegaBLAST and Blastz for alignment of ovine reads to the equine and bovine genomes. PatternHunter would perform much better if it were able to effectively utilise soft masking. A range of programs and parameter settings can be quickly surveyed with datasets appropriate to the particular objective. The optimal balance between yield and specificity of positioning chosen will depend on the subsequent use of the results.

## Methods

### General description of the method

The objective of the general methodology we present here is to compare a number of alignment programs to maximise the number of sequences generated as part of a sequencing project of species A, that are correctly positioned on the already assembled genome of a related species B. A set of paired-end reads must be obtained for species A as part of the genome sequencing project. Then the paired-end reads for species A can be used to optimise the choice of DNA sequence alignment program and parameters to align all unpaired end reads to the framework genome B, thus enabling rapid and accurate construction of sequence contigs for species A. The general approach is as follows; with the selected DNA sequence alignment programs and a range of parameters position the paired-end reads from species A on to the genome of species B. Then count the total number of sequences positioned by each program and set of parameters and calculate the number of sequences that retain the original positional relationship of paired end reads within species A within the genome of species B. Plot the results as in Figure 1 and determine the program and parameters that achieved the required balance between the total number of sequences positioned and the false positive and false negative rates. Position the remaining sequences using the chosen program and parameters.

### Mapping BAC-end sequences to the equine and bovine genome assemblies

The set of ovine BESs downloaded from GenBank were filtered as described previously [13] and "soft" masked at the most sensitive settings of RepeatMasker [17] using a repeats database built from the ovine genome sequence reads (unpublished data, personal communication Alan McCulloch). The ovine BESs were aligned to the equine genome assembly (build Equcab1.0) and bovine genome assemblies (builds Btau3.0 and Btau4.0) downloaded from the UCSC genome browser website [17] using the programs and parameters described in Table 1. BES matches to the genome assemblies were assigned to the tail-to-tail category of BACs as previously described [13], otherwise they were assigned as unpaired. For all analyses if multiple matches were returned the highest scoring match was taken as the genomic position of the BES. If more than one top match had the same score, i.e. a tie, all of the positions with the same score were checked to determine if a tail-to-tail BAC was generated with the other BES. If a tail-to-tail BAC was generated from one of the tied score positions the BES was counted as contributing to a tail-to-tail BACs, otherwise the BESs from both ends of the BAC were counted as unpaired.

BLAST version 2.2.16 was obtained from the NCBI website [18]. Blastz version 2004-Dec-22 was downloaded from the Miller laboratory website [19]. PatternHunter 2.0 was down-loaded from the Bioinformatics Solutions Inc. website [20].

### Calculation of the theoretical curve

For a given number of positioned BESs, the theoretical maximum curve estimates the expected percentage of total BESs that are correctly positioned in a tail-to-tail pair. Assuming all BESs are correctly positioned on the genome, the theoretical maximum curve was calculated using formula (1) below for values of the number of BESs with positions(*x*) ranging between 0 and 100%(1)

*X *= number of BESs with positions

*t *= Total number of BESs

*y *= Expected number of total BESs that are correctly positioned in a pair

Built into this approach is the assumption that positioning one BES correctly is independent of positioning the other end of the BAC, or of positioning it correctly. Where this is not true the number of BACs with both ends correctly positioned may exceed the theoretical maximum curve by a small amount.

The percentage of BACs in tail-to-tail configuration is always expressed as a percentage of the total BACs in the dataset, not as a percentage of BACs with at least one BES positioned.

### Calculation of false positive and false negative rates

The false positive and false negative rates as shown in Table 3 correspond to the proportion of BESs falsely predicted to be in the tail-to-tail configuration, and the number of BESs for which no position was found on the framework genome. Assuming all BESs were positioned correctly the false positive rate is calculated using formula (2) below.(2)

*o *= number of BESs actually in tail-to-tail BACs

*e *= number of BESs predicted to be in tail-to-tail BACs

## Abbreviations

BAC: Bacterial Artificial Chromosome; BES: BAC end sequence.

## Authors' contributions

AR carried out the majority of the analyses. WB and SMcW carried out some of the analyses, BPD conceived, designed and coordinated the study and drafted the manuscript. All authors drafted, read and approved the final manuscript.
